# Impact of vitamin D deficiency on iron status in children with type I diabetes

**DOI:** 10.1038/s41598-024-61559-5

**Published:** 2024-06-06

**Authors:** Eman A. M. Moslhy, May M. M. Tadros, Rasha A. Thabet, Eman H. A. Hemida, Amani F. H. Noureldeen

**Affiliations:** 1 Clinical Laboratory, National Nutrition Institute, Cairo, Egypt; 2 Biochemistry Department, Faculty of Science, Ain Shams University, Cairo, 11566 Egypt; 3 Pediatrics Department, Faculty of Medicine, Ain Shams University, Cairo, 11566 Egypt; 4 Ain Shams Specialized Hospital, Cairo, 11568 Egypt

**Keywords:** Biochemistry, Biological techniques, Diseases, Health care

## Abstract

Vitamin D deficiency (VDD) and anemia are both public health nutrition concerns. An association between VDD and anemia has been suggested in various healthy and diseased populations. The current study aimed to elucidate the effect of VDD on iron status in children with type I diabetes mellitus (T1DM). The study recruited two groups of children with T1DM: control group comprised of 38 T1DM children with sufficient vitamin D (> 30 ng/ml) and a case group, consisted of 52 T1DM children with VDD (< 20 ng/ml). Both groups had comparable gender, age, BMI, and disease duration. The laboratory measurements included analysis of blood indices, markers of iron metabolism, hepcidin and inflammatory markers included interleukin 6 (IL-6) and C-reactive protein (CRP). Compared to control group, T1DM children with VDD differs specifically in terms of some markers of blood indices, such as decreased hemoglobin and increased red blood cell distribution width. Moreover, decreased serum iron, ferritin, total iron-binding capacity and transferrin along with elevated inflammatory markers were observed in case group. Results of the study indicated that VDD had increased the risk of iron deficiency anemia in children with T1DM as well as inflammatory related anemia. Furthermore, in T1DM children, VDD had raised the incidence of both absolute and functional iron deficiency, with greater incidence of the former. This study may indicate that VDD may be a risk factor that may worsen iron deficiency anemia in T1DM.

## Introduction

Type I diabetes mellitus (T1DM) is brought on by the autoimmune destruction of endocrine pancreatic β-cells^1^. It affects an estimated of 1,211,900 children and adolescents under the age of 20 years worldwide. The incidence rate of diabetes in the Middle East and North Africa region is 8 per 100,000 population^2^. In the last few decades, much evidence suggested an intriguing link between VD homeostasis and immune responses^3^. Vitamin D (VD) has been recognized as a contributing factor to DM^4^. Data suggested that VD may be involved in the pathogenesis of T1DM due to its involvement in the modulation of the immune system and in the regulation of cell proliferation and differentiation^5^.

Iron-deficiency anemia is the most common form of anemia^6^. Children with T1DM, in early stages, have been found to be at increased risk for iron deficiency, resulting in anemia^7^. VD deficiency and anemia are both clinical conditions that coexist. The biological plausibility of this association is supported by evidence that VD regulates both hepcidin production and hepcidin-ferroprotin axis in macrophage^8^.

Our interest in studying and investigating individuals with VDD and its correlation with diabetes^9^ has prompted us to elucidate its impact on iron status and anemia in children with T1DM.

## Methodology

This case–control study was approved by the Research Ethics Committee, Faculty of medicine, Ain Shams University (code number FMASU UNIV 11/2022). The recruited children were selected from Pediatric Diabetes Clinic, Pediatric Department, Faculty of Medicine, Ain Shams University. Blood samples were collected from children after signing an informed consent by their parents or relatives. T1DM disease was diagnosed following the criteria described before^10^, including random blood sugar (200 mg/dl), glycated hemoglobin on two separate tests** = **6.5% or higher and fasting blood glucose = 126 mg/dL.

### Subjects

The study included 90 children with T1DM. They were classified into two groups: 38 children with sufficient VD served as a control group and another group of children (n = 52), matched for both age and sex, with deficient VD. The age of the recruited T1DM children (diagnosed for at least one year) ranged from 1 to 12 years. General examinations with special emphasis on weight, height measurements and calculation of body mass index (BMI) were done. The exclusion criteria were infection(s) in the last 4 weeks, congenital malformation, hemoglobinopathy, oncological disorders and iron supplementation in the last 6 weeks and/or blood transfusion in the last 6 months. All participants fulfilling the inclusion criteria were subjected to full medical history with special emphasis on history of diabetes (age at onset, duration of diabetes), family history of diabetes, hypertension, cardiovascular or renal diseases, and suggestive history of diabetic complications.

### Sample collection

Blood sample was withdrawn from each child into EDTA for complete blood count (CBC) measurement, performed using fully automated blood counter (Advia 360, Siemens). Results were interpreted according to special age and sex. Another part of the blood was collected in plain tube, sera were separated and divided into aliquots, kept at – 80 °C pending assay. Sera were used for the following determinations:

(1) Quantitative determination of human 25-OH Vitamin D2/3 using the competitive immunoassay technique, described in the employed kit provided by Epitopi Diagnostic, Inc- Germany. The kit sensitivity was 7.416 ng/ ml. Cases with more than 30 ng/ml was considered to have sufficient vitamin D.^11^

(2) Iron metabolism markers, including:

a- Serum iron determined by BT–analyzer 1500 (Biotecnica instruments–DL-Autoanalyzer) using a kit provided by Dialab–Australia. The kit sensitivity was 5 µg/dl and no interference up to bilirubin 60 mg/dl, hemoglobin up to 100 mg/dl, triglycerides 2000 mg/dl, copper 200 mg/dl and zinc 400 mg/dl.
b- Serum ferritin by double–antibody enzyme immune assay using ELISA kit purchased from Perkin Elmer–American Company. The kit sensitivity was 5.0 ng/ml. Absolute iron deficiency (AID) was defined when ferritin is < 12 µg/l in patients less than 5 years of age or when ferritin is < 15 µg/l in patients ≥ 5 years of age.
c- Serum total iron binding capacity (TIBC) determined by Chemelex, S.A, Poland Ind. Can Castells. C/Industry, Nave J.
d- Serum transferrin saturation (TSAT) was determined by a double–antibody enzyme immune assay using ELISA kit provided by Glory Science–China origin. The sensitivity of the kit was 0.1 g /L.
e- Serum transferrin (TR) was determined by a double–antibody enzyme immune assay using ELISA kit purchased from Glory Science–China origin.
f- Serum vitamin B12 was determined by a double-antibody enzyme immune assay using ELISA kit purchased from Glory Science, China origin. The sensitivity was 1.0 pg/ml.

(3) Hepcidin and inflammatory markers:

a- Serum hepcidin was determined by a double–antibody enzyme immune assay technique, using ELISA kit provided by Glory Science Co., China. The sensitivity of the kit was 1.0 ng/ml.
b- Serum interleukin-6 (IL-6) was determined in human serum by a sandwich ELISA kit provided by SinoGene Clone Biotech–China. The sensitivity of kit was 0.1 ng/ml.
c- Serum C-reactive protein (CRP) was determined using agglutination method employing assay kit provided by GPL**,** Germany.

### Statistical analysis

SPSS version 23.0 for windows (SPSS Inc, USA) was used for all analyses. To screen the data, normality tests, missing values, and outliers were checked. The Shapiro–Wilk and Kolmogorov–Smirnov tests were used to determine normality. Data are presented as mean with standard deviation (SD) for normally distributed variables, or median and interquartile range (IQR) for abnormally distributed variables. Comparison between groups were made using independent T- test for normally distributed variables and the Mann–Whitney U test for non-normally distributed variables. *P* value smaller than 0.05 was considered statistically significant.

### Ethical approval

The ethical approval was issued from the Research Ethics Committee (REC), Faculty of Medicine, Ain Shams University, under the code number FMASU UNIV 11/2022. The FMASU REC is organized and operated according to the guidelines of the International Council on Harmonization (ICH) Anesthesiology and the Islamic Organization for Medical Sciences (IOMS), the United State Code of Federal Regulations, and operates under Federal Wide Assurance No. FWA 000017585. The REC does not declare the names of its members according to the University and REC’s Standard Operating Procedures. All experiments were performed in accordance with the relevant guidelines and regulations.

## Results

### Characteristics of T1DM participants with sufficient and deficient VD

Two groups were included in this study: a control group (T1DM children with sufficient VD) representing 42% of the recruited children and a case group (T1DM children with deficient VD) representing 58% of the included children. Almost equal sex distrbution in the two groups was found with no statistical significances. Further, age, weight, height, body mass index (BMI) and duration of the disease were significantly comparable between groups (Table 1).

**Table 1:** Characteristics of T1DM participants with sufficient and deficient VD.

Parameters	Groups		
	Sufficient VD (n = 38)	Deficient VD (n = 52)	*P *Value
Gender			
Female	17.0 (44.5%)	28.0 (58.3%)	0.4
Male	21.0 (55.3%)	24.0 (46.2%)	
Age (years)*	9.0 (4.5–11.0)	10.0 (7.0–11.8)	0.2
Weight (kg)**	28.4 ± 9.69	28.6 ± 8.98	0.5
Height (m)**	123.4 ± 20.44	125.4 ± 16.37	0.2
BMI (kg /m^2^)**	18.1 ± 2.75	17.9 ± 3.75	0.4
Duration of disease (years)*	1.6 (1.0–4.0)	2.0 (1.3–4.8)	0.3
Vitamin D ()	42.3 ± 11.4	9.1 ± 4.18	0.0

*Data are presented as median (inter quartile range, IQR), **Data are presented as mean ± standard deviation (SD).

*P* value < 0.05 indicates significant, *P* value > 0.05 indicates non-significant.

### Markers of blood indices in T1DM participants with sufficient and deficient VD

Laboratory measurements for blood indices (Table 2) indicated significant reduction in the mean value of Hb in VD deficient children compared to control group, although the percentage of children with abnormal low Hb concentration (< 11 g/dl)^12^ in control (23.6%) was higher than that in the case group (17.4%). In addition, results indicated elevated median values of RDW in the case group relative to their matched values in control. It was observed that 78.8% of children in the case group were presented with elevated values in contrast to 34.2% in VD sufficient control group. Meanwhile, no significant difference was noted between the two groups for the median values of the red blood cells (RBCs) and white blood cells (WBCs) counts as well as the mean values of each of MCV and MCHC, which were within normal values. Furthermore, no significant association between blood indices and vitamin D were observed in either two groups Tables (1S and 2S).

**Table 2:** Markers of blood indices, iron metabolism and inflammatory markers in T1DM participants with sufficient and deficient VD.

	Parameters	Groups		
		Sufficient VD (n = 38)	Deficient VD (n = 52)	*P* value
Blood indices	RBCs (10^6^/μl)*	4.1 (3.8–4.6)	4.5 (3.5–4.1)	0.206
	Cases with abnormal values (low)	23.0 (60.5%)	36.0 (69.2%)	
	Hb (g/dl)**	12.3 ± 1.42	12.0 ± 0.822	0.003
	Cases with abnormal values (low)	9.0 (23.6%)	9.0 (17.4%)	
	RDW (%)*	14.4 (13–14.7)	15.0 (14.7–15.9)	0.000
	Cases with abnormal values **(**high**)**	13.0 (34.2%)	41.0 (78.8%)	
	MCV (fl)	83.1 ± 4.6	81.6 ± 3.2	0.692
	MCHC (g/dl)	32.9 ± 1.4	31.4 ± 1.1	0.520
	WBCs (10^9^/L)*	7.0 (5.17–8.0)	8.0 (5.7–10.0)	0.110
	Cases with abnormal values:			
	low	6.0 (15.7%)	4.0 (7.6%)	
	High	–	6.0 (11.5%)	
Iron metabolism markers	S. iron)µg/dl)**	61.3 ± 33.73	54.8 ± 21.38	0.006
	Cases with abnormal values (low)	1.0 (2.6%)	4.0 (7.69%)	
	Ferritin (ng/ml)*	26.5 (17.0–43.25)	19.0 (13.0–38.3)	0.050
	Cases with abnormal values **(**low)	1.0 (2.6%)	2.0 (3.8%)	
	TIBC **(**µg/dl)**	299.2 ± 30.89	279.1 ± 21.91	0.030
	Cases with abnormal values	–	–	
	TRSAT)g/L)*	0.8 (0.7—0.9)	0.8 (0.7–0.8)	0.322
	Cases with abnormal values	–	–	
	TR (g/L)*	0.3 (0.2—0.4)	0.2 (0.1–0.4)	0.000
	Cases with abnormal values	–	–	
	Vitamin B12 (pg/ml)*	23.7 (9.5—25.35)	23.0 (9.2–25.4)	0.830
	Cases with abnormal values	–	–	
Hepcidin and inflammatory markers	Hepcidin (ng/ml)**	12.6 ± 1.93	12.4 ± 3.10	0.123
	Cases with abnormal abnormal values (high)	20.0 (52.6%)	35.0 (67.3%)	
	IL-6 (ng/L)*	7.0 (5 -10)	8.5 (5 -13)	0.410
	Cases with values (high)	15.0 (39.5%)	29.0 (55.7%)	
	CRP (mg/L)*	6.0 (6–8)	8 0.0 (6–12)	0.030
	Cases with abnormal values (high)	12.0 (31.5%)	26.0 (50.0%)	

*S. iron* serum iron, *TIBC* total iron binding capacity, *TRSAT* transferrin saturation, *TR* transferrin.

*Data are presented as median. **Data are given as mean ± standard deviation (SD).

*P* value < 0.05 indicates significant, *P* value > 0.05 indicates non-significant.

### Markers of iron metabolism in T1DM participants with sufficient and deficient VD

Analysis of iron status parameters revealed significantly decreased mean values of serum iron, ferritin, TIBC and transferrin in T1DM children with deficient VD relative to those with sufficient VD. Other parameters, such as TRSAT and vitamin B12, showed no significant variations between the two studied groups (Table 2).

### Hepcidin and inflammatory markers in T1DM participants with sufficient and deficient VD

Although the results in this study displayed no significant differences in the mean values of hepcidin between the two studied groups, however, it was abnormally elevated in some children with T1DM both with sufficient and deficient VD. Greater incidence in cases with deficient VD (67.3%) confirmed suppression action of VD deficiency on hepcidin (Table 2).

In the current study, while the median value of IL-6 did not illustrate significant variations between the included groups of children, the C-reactive protein median values showed significant elevation in T1DM children group with deficient VD compared to their matched values in the control (Table 2).

### Percent of inflammatory and iron deficiency anemia (IDA) in T1DM children with sufficient and deficient VD

**Figure 1:**
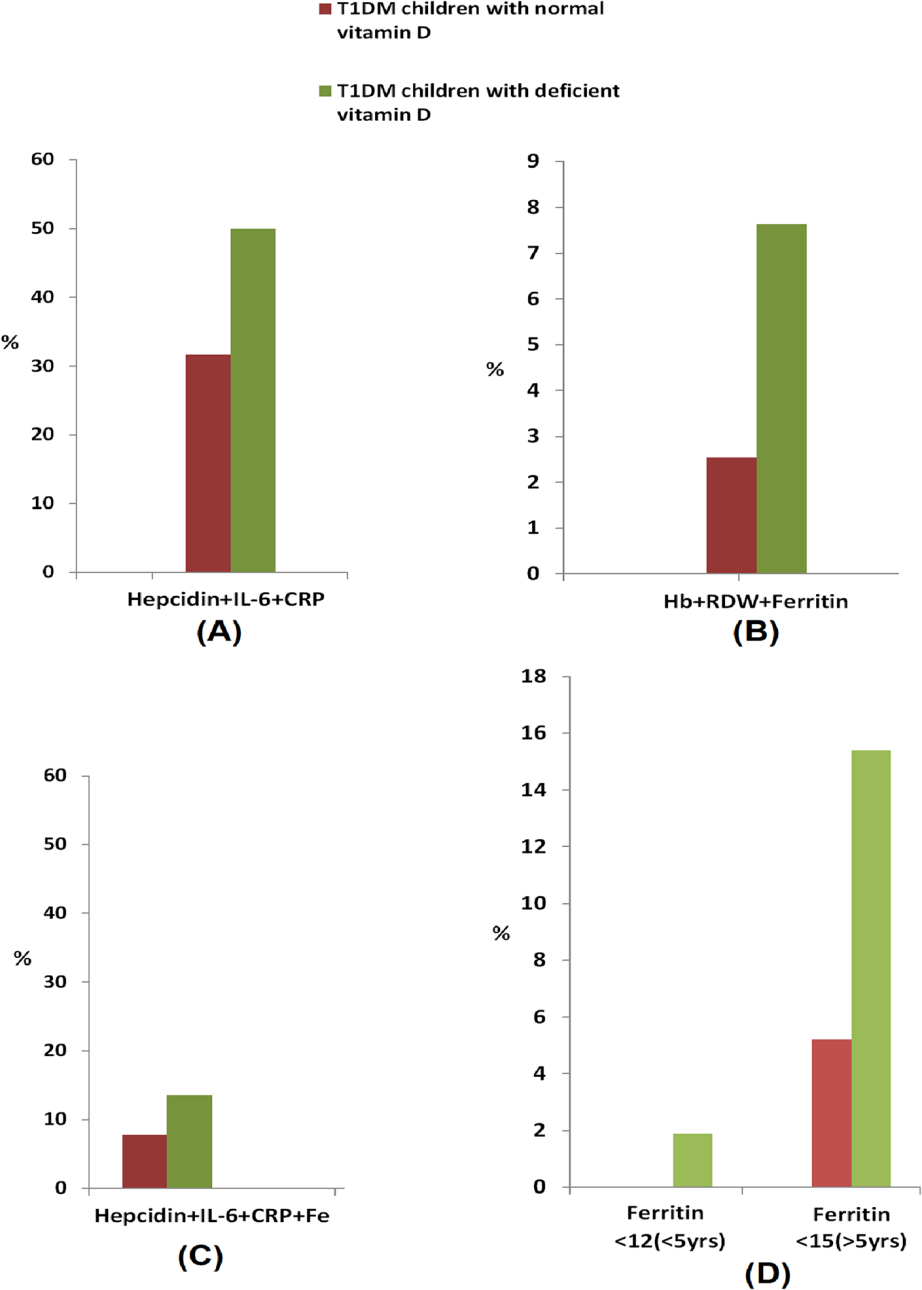
The percent of (**A**) inflammatory related anemia in sufficient and deficient VD groups; (**B**) iron deficiency anemia (IDA) in T1DM children in sufficient and deficient VD groups; (**C**) cases with FID in T1DM participants with sufficient and deficient VD; (**D**) cases with absoluteiron deficiency (AID) in T1DM children with sufficient and deficient VD.

As obviously shown from Fig. 1A, the percentage of inflammatory related anemia was higher in TIDM children with deficient VDD compared to children with sufficient VD. Moreover, the incidence of iron deficiency anemia (characterized by a decrease in both Hb content and ferritin levels < 12 µg/ml with increase in RDW) was higher in children with T1DM and deficient VD (7.7%) compared to incidence in control group of children with sufficient VD (2.6%), Fig. 1B. It is worth mentioning that there were only two cases with ferritin level below normal range representing 3.8% in the VD deficient group and another two children with low normal ferritin accompanied with a decreased Hb and elevated RDW that met the criteria of IDA.

### The percentage of functional and absolute iron deficiencies (FID and AID) in T1DM participants with sufficient and deficient VD.

As indicated from Fig. 1C, FID (abnormal levels of hepcidin + IL-6 + CRP + S. iron)^13,14^ was observed in both groups of T1DM children, either with sufficient or deficient VD. The percentage of cases with FID were higher in T1DM children with deficient VD (13.5%) compared to cases in the control group (7.7%). On the other hand, in the absence of infection and/or acute inflammation (CRP-concentration ≤ 10 mg/l) and according to the World Health Organization^6^, AID was defined when ferritin is < 12 µg/l in patients less than 5 years of age or when ferritin is < 15 µg/l in patients ≥ 5 years of age^15^. Higher percentage values of AID were observed in T1DM with deficient VD in children with age less than 5 years (1.9%) compared to T1DM children’s cases with normal vitamin D (0.0%). Furthermore, AID was detected in 15.4% of children with age ≥ 5 years who are suffering from deficient VD relative to 5.2% of age matched T1DM children with sufficient VD (Fig. 1D).

## Discussion

Currently, vitamin D deficiency resulted in decreased Hb concentration and elevated RDW in TIDM children, however other blood indices showed no significant variations. Some studies indicated no change in blood indices in TIDM children compared to control children^16,17^ except for Hb and RDW. Furthermore, others indicated that some blood indicators are different and changed at late disease duration^18^. RDW is a standard complete blood count that measures the variations in either RBCs size or volume. It could also be used to determine the causes of anemia^7^. The inclusion of RDW in the complete blood count assessment has made diagnosing certain anemia easier, especially those that are microcytic^19^. Elevated RDW helps in the diagnosis of early nutritional deficiency such as iron and vitamin B12, as it becomes elevated earlier than other RBCs parameters^20^. Increased RDW occurs in cases with iron deficiency anemia as well as in anemia with inflammation, while it is being higher in the former^21^. Hyperglycemia has multiple effects on RBCs, comprising decreased deformability, a decreased life span and glycation of Hb^22^. It is associated with multiple micro-vascular complications leading to nephropathy, retinopathy, and neuropathy. Mounting evidence suggested that red blood cells alterations are both a cause and a consequence of disturbances related to DM-associated complications. Importantly, a significant proportion of DM patients develop varying degrees of anemia of confounding etiology, leading to increased morbidity. In chronic hyperglycemia, RBCs display morphological, enzymatic, and biophysical changes, which in turn prime them for swift phagocytic clearance from circulation^7^. RDW but not Hb level, was found to be higher in T1DM children compared to normal children^20^. The current study indicated a significant negative association between RDW and Hb in sufficient vitamin D group. In deficient vitamin D children, RDW was negatively correlated with both Hb and serum iron, indicating a strong association between RDW and anemia, especially in vitamin D deficiency (Tables 1S and 2S).

Our study indicated decreased serum iron, ferritin, TIBC and transferrin in cases with deficient VD compared to control group with sufficient VD. Although we failed to obtain a significant association between vitamin D and parameters of iron metabolism in both groups of children, the obtained results indicated that children with VDD may be at increased risk for iron deficiency. Noteworthy to indicate that some children in both studied groups had low serum iron, however the percentage was higher in deficient VD cases (7.6% of cases with VD deficiency and 2.6% in control with sufficient VD). A previous study^16^ indicated that T1DM children had normal range for iron metabolism parameters and vitamin B12, the study did not indicate significant difference with the control healthy children, while ferritin and TIBC were lower in T1DM group. It would be ascribed to indicate that the duration of disease was not correlated with each of Hb, TIBC, serum iron, vitamin B12 and ferritin indicating regular control and healthy nutrition Tables 1S and 2S^17^.

Our results illustrated no significant difference in the mean value of hepcidin between the two studied groups. However, the incidence of elevated hepcidin in some children with deficient VD was greater than the children with sufficient VD. Such action was previously observed^23–25^. Hepcidin is a systemic regulator of iron homeostasis. It is produced because of iron stimulation as well as in inflammatory states. The role of hepcidin is to stop iron in intracellular pool and decrease its gastrointestinal absorption, therefore decreasing serum iron. This is a beneficial defensive mechanism which hides iron from being used by bacteria^21^. Although the current study revealed no significant difference in the mean value of hepcidin between the two groups of children with deficient and sufficient VD, however, greater incidence in cases with deficient VD (67.3%) confirmed suppression action of VD deficiency on hepcidin secretion. Currently, while the median value of IL-6 did not illustrate significant variations between the included groups of children, the C-reactive protein median values showed significant elevation in T1DM children group with deficient VD compared to their matched values in the control. VD has been shown to promote erythropoiesis by increasing erythroid progenitor proliferation and decreasing pro-inflammatory cytokines. Additionally, VD might suppress hepcidin expression by decreasing hepcidin-stimulatory pro-inflammatory cytokines, and through direct transcriptional regulation of the hepcidin antimicrobial peptide (HAMP) gene. Decreased pro-inflammatory cytokines and hepcidin might increase iron bioavailability for erythropoiesis and hemoglobin synthesis by restoring iron recycling, preventing iron sequestration in macrophages, and removing impairments on iron absorption, and thus protecting against anemia^26^. In the present investigation, VD deficiency had raised the incidence of inflammation as indicated by elevated serum IL-6 and/ or CRP in T1DM children with deficient VD relative to T1DM children with sufficient vitamin D. Elevated serum IL-6 and CRP reached 55.8% and 50.0%, respectively, for cases with deficient VD compared to 39.5% for IL-6 and 31.6% for CRP in children with sufficient vitamin D. Furthermore, inflammatory related anemia as indicated from the increase in both hepcidin and the inflammatory markers, either IL-6 and/ or CRP, were detected in the recruited children with sufficient (31.6%) and deficient (50.0%) VD. Further, significant positive correlations between hepcidin and the inflammatory markers in both two groups of children Tables 1S and 2S were found, suggesting a strong association between inflammation and elevated hepcidin. This may also suggest a reduced state of chronic systemic inflammation in those children with sufficient VD or an ineffective erythropoiesis in T1DM children with deficient VD^27^.

It was previously observed that factors present in diabetes may increase the risk of anemia. In diabetes, anemia develops earlier than in patients with renal impairment^28–30^. As noted from the current study, vitamin D deficiency increased the incidence for anemia. A greater prevalence and risk of anemia in individuals with vitamin D deficiency was reported and have been compared with those having sufficient vitamin^27^. The authors reported 48% prevalence in vitamin D deficient group with T1DM versus 36% with normal vitamin D.

Under conditions of VD deficiency, elevated synthesis of hepcidin by hepatocytes or monocytes may increase intracellular and systemic concentrations of hepcidin and decrease membrane expression of ferroprotin (Fp) in these cells. The resulting suppression of iron export will, in turn, lead to intracellular accumulation, increased cellular ferritin, and decreased systemic levels of iron. Furthermore, decreased transcription of HAMP gene may lead to decrease the intracellular and systemic concentrations of hepcidin and concomitant the membrane expression of Fp increases. The enhancement of iron export may lead to a decrease in the intracellular iron and ferritin and an increase in the systemic levels of iron^25^.

It was previously reported that iron deficiency anemia could exist either as absolute iron AID or FID^31,32^. The current study concluded that vitamin D deficiency increased the incidence to develop both FID and AID in T1DM. Depending on the age of the recruited children, on the other hand, the incidence of AID was elevated in cases with deficient VD. However, some other investigations showed iron deficiency in T1DM without differentiating between AID and FID^33,34^. Nevertheless, some previous studies explored the existence of both two types in T1DM^35,36^. Higher incidence of AID and FID (5.7% and 47% respectively) were also previously indicated^36^.

## Conclusion

VD deficiency had increased the risk of iron deficiency as well as inflammatory related anemia in T1DM. In children with T1DM an increased incidence of both absolute and functional iron deficiency, with greater incidence of the former would be ascribed to deficiency of vitamin D. These findings may indicate that vitamin D deficiency could be a risk factor that worsen the iron deficiency anemia in T1DM. The current work may simply imply that adjusting vitamin D level could improve anemia associated with T1DM.

## Supplementary Information

Supplementary Tables.

## Data Availability

Any datasets used that support the findings of this study are available from the corresponding author upon reasonable request. A supplementary file, containing the table of correlations study between each of vitamin D, BMI, duration of disease, RDW and Hepcidin versus all studied parameters in T1DM children with sufficient VD group.
