# Supplementary material for: Impact of vitamin D deficiency on iron status in children with type I diabetes
Source: Sci Rep. 2024 Jun 6;14:12989. doi: 10.1038/s41598-024-61559-5 (PMC11156629; doi:10.1038/s41598-024-61559-5)
Supplement: Supplementary file 1 — Supplementary Tables. [file 41598_2024_61559_MOESM1_ESM.docx]

**Supplementary file**

**Impact of Vitamin D Deficiency on Iron Status in Children with Type I Diabetes**

**Eman A.M. Moslhy^1^, May M.M. Tadros^2^, Rasha A. Thabet^3^, Amani F.H. Noureldin^2,^***

^1^ Biochemistry department, Faculty of Science, Ain Shams University, Cairo, Egypt.

^2^ Biochemistry department, National Nutrition Institute, Cairo, Egypt

^3^ Pediatrics Department, Faculty of Medicine, Ain Shams University, Cairo, Egypt.

**Table 1S. Correlations study between each of vitamin D, BMI, duration of disease, RDW and Hepcidin versus all studied parameters in T1DM children with sufficient VD group**

| **Sufficient VD group** | | | | | | | | | | |
| --- | --- | --- | --- | --- | --- | --- | --- | --- | --- | --- |
| **Vitamin D** | | | **BMI** | | **Duration of disease** | | **RDW** | | **Hepcidin** | |
|  | **r** | ***P***  **value** | **r** | ***P***  **value** | **r** | ***P***  **Value** | **r** | ***P***  **value** | **r** | ***P***  **Value** |
| **Gender** | 0.135 | 0.418 | - 0.193 | 0.245 | - 0.055 | 0.74 | - 0.058 | 0.703 | - 0.123 | 0.461 |
| **Age** | - 0.198 | 0.233 | - 0.193 | 0.419 | 0.454 | 0.004 | - 0.164 | 0.324 | - 0.062 | 0.711 |
| **Weight** | - 0.243 | 0.142 | 0.211 | 0.203 | 0.439 | 0.006 | - 0.402 | 0.012 | 0.207 | 0.213 |
| **Height** | - 0.215 | 0.196 | - 0.186 | 0.262 | 0.331 | 0.043 | - 0.425 | 0.008 | 0.141 | 0.399 |
| **BMI** | 0.072 | 0.669 | - | - | 0.156 | 0.349 | 0.071 | 0.67 | 0.18 | 0.281 |
| **Duration of disease** | - 0.158 | 0.342 | 0.156 | 0.349 | - | - | - 0.274 | 0.096 | - 0.132 | 0.431 |
| **RBCs** | - 0.036 | 0.831 | 0.264 | 0.109 | 0.001 | 0.996 | - 0.24 | 0.146 | 0.119 | 0.473 |
| **Hb** | - 0.048 | 0.775 | - 0.015 | 0.931 | 0.308 | 0.06 | - 0.81 | 0.0 | - 0.135 | 0.418 |
| **RDW** | 0.118 | 0.482 | 0.117 | 0.483 | - 0.274 | 0.096 | - | - | - 0.002 | 0.992 |
| **WBCs** | 0.292 | 0.075 | 0.193 | 0.246 | - 0.008 | 0.961 | 0.368 | 0.063 | - 0.179 | 0.28 |
| **S. iron** | - 0.207 | 0.213 | - 0.186 | 0.263 | - 0.017 | 0.922 | - 0.156 | 0.351 | 0.093 | 0.577 |
| **Ferritin** | 0.269 | 0.102 | 0.501 | 0.001 | 0.139 | 0.406 | - 0.25 | 0.131 | 0.043 | 0.8 |
| **TIBC** | 0.066 | 0.695 | - 0.021 | 0.899 | - 0.026 | 0.875 | - 0.024 | 0.888 | 0.079 | 0.637 |
| **TRSAT** | 0.188 | 0.259 | 0.189 | 0.256 | 0.069 | 0.68 | - 0.165 | 0.321 | 0.209 | 0.209 |
| **TR** | - 0.343 | 0.035 | 0.114 | 0.497 | 0.344 | 0.034 | - 0.122 | 0.466 | 0.375 | 0.02 |
| **Vit.B12** | 0.088 | 0.599 | - 0.091 | 0.588 | - 0.168 | 0.314 | 0.096 | 0.566 | - 0.054 | 0.746 |
| **Hepcdin** | - 0.074 | 0.657 | 0.27 | 0.101 | - 0.077 | 0.647 | - 0.151 | 0.363 | - | - |
| **IL-6** | 0.261 | 0.114 | 0.039 | 0.816 | - 0.156 | 0.349 | 0.0 | 1.0 | 0.709 | 0.0 |
| **CRP** | 0.172 | 0.302 | 0.236 | 0.154 | - 0.181 | 0.276 | 0.041 | 0.807 | 0.802 | 0.0 |
| **Vitamin D** | - | - | 0.072 | 0.669 | - 0.158 | 0.342 | 0.118 | 0.482 | - 0.074 | 0.65 |

**Table 2S. Correlations study between each of vitamin D, BMI, duration of disease, RDW and Hepcidin versus all studied parameters in T1DM children with deficient VD group**

| **Deficient VD group** | | | | | | | | | | |
| --- | --- | --- | --- | --- | --- | --- | --- | --- | --- | --- |
| **Vitamin D** | | | **BMI** | | **Duration of disease** | | **RDW** | | **Hepcidin** | |
|  | **r** | ***P***  **value** | **r** | ***P***  **value** | **r** | ***P***  **value** | **r** | ***P***  **value** | **r** | ***P***  **value** |
| **Gender** | 0.49 | 0.730 | - 0.53 | 0.28 | 0.099 | 0.48 | - 0.14 | 0.29 | 0.18 | 0.553 |
| **Age** | - 0.101 | 0.478 | 0.121 | 0.393 | 0.333 | 0.016 | - 0.17 | 0.229 | 0.333 | 0.016 |
| **Weight** | -.0.034 | 0.812 | 0.545 | 0 | 0.241 | 0.08 | - 0.154 | 0.257 | 0.186 | 0.187 |
| **Height** | 0.090 | 0.525 | - 0.098 | 0.491 | 0.197 | 0.161 | - 0.107 | 0.451 | 0.228 | 0.104 |
| **BMI** | 0.002 | 0.987 | - | - | 0.13 | 0.357 | - 0.123 | 0.38 | - 0.038 | 0.787 |
| **Duration of disease** | 0.032 | 0.821 | 0.13 | 0.357 | - | - | - 0.239 | 0.088 | - 0.173 | 0.219 |
| **RBCs** | 0.053 | 0.707 | 0.103 | 0.229 | 0.171 | 0.224 | - 0.016 | 0.912 | 0.038 | 0.789 |
| **Hb** | - 0.066 | 0.640 | 0.137 | 0.33 | 0.254 | 0.069 | - 0.562 | 0.0 | - 0.205 | 0.144 |
| **RDW** | - 0.015 | 0.913 | - 0.121 | 0.385 | - 0.239 | 0.088 | - | - | 0.15 | 0.288 |
| **WBCs** | - 0.103 | 0.469 | 0.079 | 0.57 | - 0.152 | 0.281 | 0.249 | 0.075 | - 0.083 | 0.559 |
| **S. iron** | - 0.130 | 0.359 | 0.283 | 0.042 | 0.324 | 0.019 | - 0.396 | 0.004 | - 0.025 | 0.858 |
| **Ferritin** | - 0.100 | 0.482 | 0.413 | 0.116 | 0.106 | 0.453 | - 0.159 | 0.261 | 0.089 | 0.532 |
| **TIBC** | - 0.009 | 0.951 | 0.016 | 0.911 | 0.075 | 0.569 | - 0.047 | 0.74 | 0.246 | 0.079 |
| **TRSAT** | - 0.182 | 0.198 | - 0.045 | 0.754 | - 0.07 | 0.621 | - 0.05 | 0.72 | 0.242 | 0.084 |
| **TR** | - 0.714 | 0.000 | 0.335 | 0.015 | - 0.042 | 0.76 | - 0.275 | 0.048 | - 0.222 | 0.114 |
| **Vit.B12** | - 0.137 | 0.331 | - 0.022 | 0.877 | - 0.195 | 0.169 | - 0.003 | 0.981 | 0.289 | 0.038 |
| **Hepcdin** | - 0.006 | 0.966 | - 0.038 | 0.787 | - 0.171 | 0.219 | 0.15 | 0.288 | - | - |
| **IL-6** | 0.002 | 0.987 | 0.05 | 0.69 | - 0.217 | 0.122 | 0.192 | 0.172 | 0.715 | 0.0 |
| **CRP** | -.0.025 | 0.862 | 0.024 | 0.865 | - 0.257 | 0.066 | 0.105 | 0.46 | 0.707 | 0.0 |
| **Vitamin D** | - | - | 0.174 | 0.218 | 0.032 | 0.821 | - 0.015 | 0.913 | - 0.117 | 0.409 |
